# New Metal Shield for the Endotracheal Tube during Laser Endolaryngeal Surgery

**Published:** 2018-09

**Authors:** Nasser-Nagieb Mohamed, Mohammad-Waheed El-Anwar

**Affiliations:** 1 *Department of Otorhinolaryngology-Head and Neck Surgery, Faculty of Medicine, Zagazig University, Zagazig, Egypt. *

**Keywords:** Endotracheal tube, Ignition, Laser, Larynx, Laryngoscopy, Voice, Protection

## Abstract

**Introduction:**

To present and assess a newly designed autoclavable, reusable tube shield for an endotracheal tube (ETT) during laser laryngeal surgery.

**Materials and Methods:**

This study included patients scheduled for endolaryngeal laser surgery. A carbonated stainless-steel hemisphere shield (~1.5×0.6 mm) connected to a silk thread passing through a hole in its middle near upper edge was used. Under general anesthesia, patients were intubated orally using a small cuffed polyvinyl ETT, filling the cuff with saline. When the laser was used, the shield was applied either to the side of the tube or anterior to it, depending on the site of the laser procedure. The shield could easily be repositioned when needed during surgery.

**Results:**

In 25 cases (six cordectomy for glottic carcinomas and 19 posterior cordotomy for bilateral vocal cord paralysis), application of the shield over the ETT was easy and the shield could be easily and simply fitted in all cases, with change in position achieved easily during the work as required. The shield over the ETT could not be penetrated by the laser, regardless of the gas composition or laser energy. No intraoperative complications were encountered in any of our cases. Recovery was event-free in all patients without the need for admission to an intensive care unit.

**Conclusion:**

The newly designed autoclavable reusable stainless-steel shield designed for an ETT could be safely, easily, and effectively used during laser laryngeal surgery with low cost and easy availability. However, comparative and multicenter studies are still needed.

## Introduction

Since 1972 when Strong and Jako first reported the use of a carbon dioxide (CO_2_) laser in the human larynx (1), the laser has continued to be used in laryngeal surgery. Since then, endoscopic laser treatments have been successfully utilized in endolaryngeal microsurgery, and have gained universal acceptance (2). Fire in the endotracheal tube (ETT) is the most frequent complication of laser surgeries of the larynx (3), and can cause an airway fire that represents the most morbid complication of this procedure (4).

 Various measures have been used to minimize laser-related hazards during laryngeal laser microsurgery (5). With the increasing popularity of laryngeal laser microsurgery, anesthesiologists and surgeons inevitably need to select the safest and most efficient ETT to avoid ETT fires or combustion (6).

Various methods have been proposed to protect the ETT and larynx, including wrapping aluminum tape around the ETT, placing wet cottonoids around the ETT cuff, filling the cuff with saline, utilizing a Xomed or metal ETT, and using a Venturi (jet) method for ventilation (4). However, a foil wrapping may unravel and leave exposed parts of the ETT, while the reflective material may result in laser reflection onto the normal tissue, causing undesirable burning (7). 

Furthermore, moist cottonoids may dry out, becoming quite flammable and allowing pieces to be lost that could be easily aspirated. Although a metal ETT ensures complete safety during laser therapy (5,7), the metal ETT is usually cuffless and bulky, meaning that successful intubation is more difficult. 

Venturi ventilation is helpful, particularly in pediatric patients, but needs special equipment and it is associated with many serious complications (8).

The optimal reconstructed ETT should be designed to avoid perforation and ignition with subsequent inhalation of combustion products that are very strong lung irritants, regardless of gas composition or laser energy, and should have a low price (1,2).

This study aimed to present and evaluate a newly designed autoclavable reusable tube shield for the ETT during laser laryngeal surgery.

## Materials and Methods

This prospective cohort study included patients scheduled for endolaryngeal laser surgery between May 2013 and January 2016 at the otorhinolaryngology head and neck surgery department of Zagazig University hospitals. The study protocol was approved by the Institutional Review Board, and the investigators obtained written informed consent from all participants. Patients who were refused surgery or judged unfit for general anesthesia were excluded from the study. All patients were treated using an endoscopic diode laser for either posterior cordotomy in bilateral abductor palsy (as described by Mohamed et al.) (9) or by laser excision of glottic or ventricular tumor, tailored according to tumor extension.

**Fig 1:**
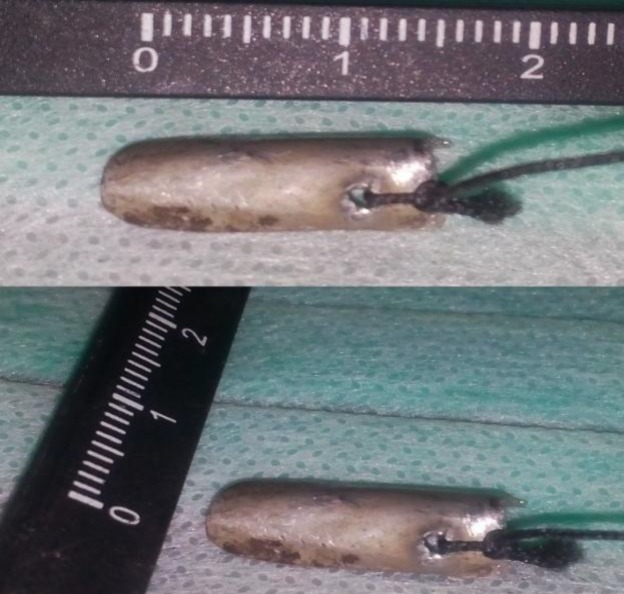
the designed carbonated stainless steel shield connected to a silk thread passing through a hole in its middle near upper edge of nearly 1.5 cm length and 0.6 cm width

The shield consisted of a carbonated stainless-steel hemisphere connected to a silk thread passing through a hole in its middle near upper edge. The shield was fabricated to be nearly 1.5 cm in length and 0.6 cm in width (Fig.1).

**Fig 2:**
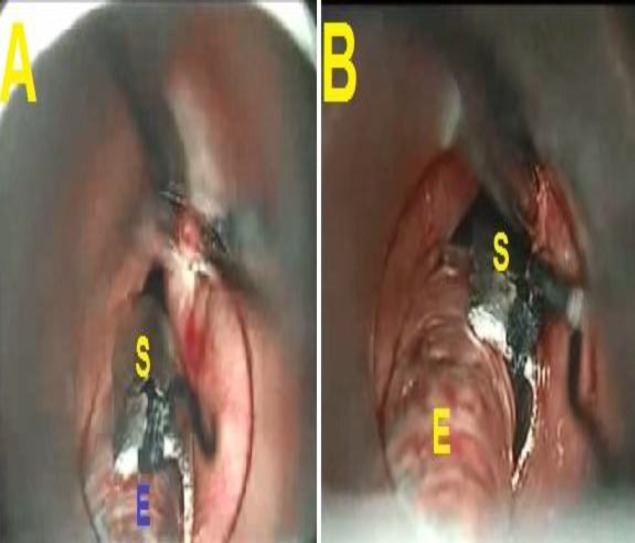
The shield (S) was applied either anterior (A) to endotracheal tube (E) or to the side (B) of the tube during laser work.

Under general anesthesia, patients were intubated orally using a small-sized cuffed polyvinyl ETT, filling the cuff with saline. Then, the designed hemisphere stainless-steel shield was applied to protect the ETT when the laser was used. The shield could be applied either to the side of the tube or anterior to it, depending on the site of the laser operation (Fig.2). 

The shield can easily be repositioned when needed during surgery. The curve in the shield allows it to be self-fitted to any size of the used ETT, and this stabilizes the shield in its desired position during the laser procedure. Also, the curvature of the shield gives extra protection to the tube as it leaves some insulating air space between the shield and the ETT.

Saline-moistened surgical cottonoid was kept in the subglottic area to protect the ETT cuff. A suitable-sized laryngoscope was inserted and suspended to expose the larynx.

A surgical microscope (Zeiss) with a 400-mm lens was utilized for appropriate magnified view of the larynx. After initial endoscopic assessment of the lesion, the shield was applied by the surgeon and the laser was used. The shield also helped to lateralize the lesion when large, making its excision easier. After the end of the endoscopic laser procedure, the shield could be removed by traction on its silk.

## Results

**Twenty-fiv** cases undergoing trans-oral laser endoscopic surgery were included in this study, consisting of six (24%) cases of cordectomy for glottis carcinomas and 19 (76%) posterior cordotomy for bilateral vocal cord paralysis. The ages of the participants ranged from 31 to 73 years (mean 47±14.45 years). Eleven (44%) males (including all cancer cases) and 14 (56%) females were included (Table.1).

**Table 1:** basic and demographic information for patients

**Surgical type**	Cordectomy for glottis carcinomas	6 (24%) patients
	Posterior cordotomy for bilateral vocal cord paralysis.	19 (76%) patients
**Age**	Range	31 to 73 years
	Mean	47 ± 14.45
**Sex**	Male	11 (44%)
	Female	14 (56%)

No patients required tracheostomy before or after the procedure. Because the anesthetists used conventional ETT during intubation, they did not encounter any difficulties. Application of the shield over the ETT was easy, and it could be easily and simply fitted in all cases, with facile change in its position during the work, as required. The shield over the ETT was not penetrated by the laser, regardless of the gas composition or laser power. Intraoperative complications such as tube ignition, fires, or combustion were not encountered in any of our cases. Recovery was event-free in all patients without the need for intensive care unit admission.

Patients began oral feeding 4 hours post surgery, and left the hospital on the next day.

## Discussion

The most morbid and serious complication of laryngeal laser surgery is ETT fire, which may be reported even with the use of special laser ETT and laser safety precautions (6). Sesterhenn et al. analyzed the data from 15 reported incidents of ETT fire, and questioned whether special laser ETT use is really justified considering its high cost and when even laser safety tubes offer no guarantees against accidental tube fires (6). The registered incidence of ignition during microlaryngeal laser surgery ranges from 0.14% to 1.5% (10), and ETT fire was reported with a number of different tube materials (11).

In an attempt to avoid the most serious complication of endolaryngeal laser surgery, we designed the described stainless-steel tube shield. Stainless steel is the safest material used that can resist the effect of the laser, while carbonation of the shield prevents unwanted laser beam reflections.The newly designed hemisphere shield can be applied and fitted to the side of the tube on one laryngeal side, with proper protection to all exposed areas of the ETT to laser beam (anterior, side of work and posterior). The shield can also be fitted anterior to the ETT, thus protecting the anterior and sides of the ETT. Therefore, the shield position depends on the site of the laser procedures, and it can be easily relocated during surgery. The curve of the shield allows it to be fitted to an adult ETT of any size. The use of a shield was remarkably beneficial in excising malignant tumors extending posteriorly near the tube and in posterior cordotomy. It enabled us to work with more confidence in this high-risk posterior area in close contact to the tube, which enabled us to achieve complete and safe excision of posterior extension of tumors. As an extra safety precaution, the shield was secured using a silk thread through a hole in its upper part.

This carbonated stainless-steel shield is autoclavable, making it economically very efficient. In addition, the shield can fit to all adult sizes of ETT meaning that a set of shields is not required. Furthermore, the new concept of applying a carbonated stainless-steel shield to conventional polyvinyl ETT obviates the need to use a special laser ETT, which reduces the cost of laser surgery because a conventional polyvinyl tube can be used and protected by the shield. The shield might be valuable even from an economic point of view because it is autoclavable, and therefore reusable. It also has no cuff or valve that would need to be checked following sterilization after each use for any dysfunction.

Because the weakest ETT point is usually in the cuff area, which is the major source of ETT fires or ignitions (6,12–14), the cuff should be filled by saline and protected by surgical cottonoid moistened with saline and positioned in the subglottis region during surgery (6,9,12–14), either with laser-resistant ETT or the described shield. Using the new stainless-steel shield with the ordinary cuffed polyvinyl ETT protects the operative field from inflammable anesthetic gases and also obviates the difficulty of inserting less flexible and larger metal ETTs that might have no cuff and may fill the operative field.

The laser shield for the ETT is not reflective and does not need advance preparation (as in foil wrapping). It is easy to place in the preferred position and to change its site. It is separated from the tube and so does not interfere with tube admission and does not increase thickness of the tube during intubation. These characteristics are attractive to the surgeon during laser surgery of the larynx. Additionally, this simple and easily fabricated shield can be readily available, and can thus be considered a valuable safety tool in endolaryngeal laser surgery, safeguarding against the horrifying complication of combustion. However, comparative studies between the described shield and laser-resistant ETT are recommended, and also multicenter studies are also needed. In addition, we agree with most previous studies on the safety of laser-resistant ETT, that even when using special laser tubes or newly described metal shield, other safety measures should be followed. We believe that safe laser surgery of the larynx can be conducted using the newly designed laser-resistant shield compared with the usual types of ETT.

## Conclusion

The newly designed autoclavable reusable stainless-steel shield for the ETT can be safely, easily, and effectively used during laser microlaryngeal surgery with low cost and easy availability. However comparative studies with laser-resistant ETT are needed.
